# A complete classification of epistatic two-locus models

**DOI:** 10.1186/1471-2156-9-17

**Published:** 2008-02-19

**Authors:** Ingileif B Hallgrímsdóttir, Debbie S Yuster

**Affiliations:** 1 Department of Statistics, University of Oxford, 1 South Parks Road, OX1 3TG, UK; 2 Department of Mathematics, Columbia University, New York, NY 10027, USA; 3 DIMACS Center, Rutgers University, 96 Frelinghuysen Road, Piscataway, NJ 08854, USA

## Abstract

**Background:**

The study of epistasis is of great importance in statistical genetics in fields such as linkage and association analysis and QTL mapping. In an effort to classify the types of epistasis in the case of two biallelic loci Li and Reich listed and described all models in the simplest case of 0/1 penetrance values. However, they left open the problem of finding a classification of two-locus models with continuous penetrance values.

**Results:**

We provide a complete classification of biallelic two-locus models. In addition to solving the classification problem for dichotomous trait disease models, our results apply to any instance where real numbers are assigned to genotypes, and provide a complete framework for studying epistasis in QTL data. Our approach is geometric and we show that there are 387 distinct types of two-locus models, which can be reduced to 69 when symmetry between loci and alleles is accounted for. The model types are defined by 86 circuits, which are linear combinations of genotype values, each of which measures a fundamental unit of interaction.

**Conclusion:**

The circuits provide information on epistasis beyond that contained in the *additive × additive, additive × dominance*, and *dominance × dominance *interaction terms. We discuss the connection between our classification and standard epistatic models and demonstrate its utility by analyzing a previously published dataset.

## Background

The genetic dissection of complex traits is at the center of current research in human genetics. Complex traits are caused by multiple susceptibility genes and environmental factors, and mounting evidence from both human genetics and model organisms suggests that *epistasis *(gene × gene interaction) plays an important role [1,2]. Although the need to consider epistasis when mapping complex trait loci has been discussed by several authors [3-6], most statistical methods used in gene mapping, be it case-control association studies, quantitative trait loci (QTL) mapping, or linkage analysis, are based only on measures of marginal effects at individual loci and do not consider epistasis. Due to recent advances in genotyping technology many large case-control genome-wide association studies [7] have recently been completed, and there has been renewed interest in two-locus disease models and two-locus tests for association [8-11]. The application of two-locus models also arises in expression QTL mapping where thousands of gene expression traits are mapped with linkage analysis and it is imperative to study gene interactions [12].

Ideally, a test for epistasis between two loci *A *and *B *should test for *biological interaction*, or non-independence between the effects of locus *A *and locus *B*. Loci *A *and *B *are considered independent if the effect of the genotype at locus *A *does not depend on the genotype at locus *B*. This biologically motivated concept has been formalized in a variety of ways by different communities seeking simple, mathematically convenient definitions. In the statistical genetics literature the term epistasis is typically taken to mean that the effects at loci *A *and *B *are not additive (the "effect" of a locus is defined in terms of the statistical model used [13]). For a further discussion on epistasis see [14-16]. Fisher [17] considered a linear model for the contribution of different loci to a quantitative trait and used the term *epistasy *to describe a departure from additivity. In linkage analysis based on variance component models, a model without epistasis is a model in which all dominance variance components are zero. In case-control association studies of dichotomous traits it is common to use logistic regression, and additivity is measured in the log-odds of disease for a genotype [18]. A new test for epistasis was recently suggested in [10], it tests for departures from linkage disequilibrium (LD) in the cases, which is equivalent to testing for departure from additivity of the log penetrance values (i.e. departure from a multiplicative model for the two-locus penetrances). These tests all test for departure from additivity on a particular scale, but if an additive model is rejected they provide no information on the type of interaction present. Furthermore, it is not clear what the biological meaning of the interaction is.

With each of the nine two-locus genotypes we associate a *genotype value*. In the case of a dichotomous phenotype the genotype value can e.g. be the penetrance associated with the genotype, the logarithm of the penetrance, or the logarithm of the odds ratio. In the case of a quantitative trait a natural choice for the genotype value is the expected phenotype value of individuals with that genotype (sometimes called *measured genotype*). We will consider epistasis to be any deviation from additivity of the genotype values. This is consistent with the definition of epistasis given in [13], both for quantitative and dichotomous traits. In this paper we provide a framework within which one can study and classify the types of epistasis possible between two biallelic loci. Our results are based on recent work of [19] who provide a rigorous geometric approach to epistasis in the haploid case. We extend their results to the diploid case, and characterize all possible patterns of physical interactions among the 9 possible genotypes in the two locus case, showing that there are 387 classes of models that fall into 69 symmetry classes. We discuss the meaning of the different types of interaction and show how the interaction pattern can be effectively measured and visualized.

In genetic analysis it is common to test not only for departure from additivity, but also for whether the data fits a particular two-locus model (e.g. recessive or dominant). We discuss the models that are frequently used and show how they relate to the classification given here. In order to study a wider class of two-locus models [20] enumerated all two-locus, two-allele, two-phenotype disease models with penetrance values 0 or 1 for the nine possible phenotypes. There are 512 such models, which can be reduced to a list of 50 models after allowing for symmetry between alleles, loci and affection status. We classify models with continuously varying penetrances, overcoming the difficulty they highlight in their paper, and show that in fact their 50 models fall into 29 of the 69 symmetry classes.

We introduce the mathematical concepts used to derive the 387 classes of two-locus models and demonstrate on a real dataset how the shapes can be used to classify pairs of loci and identify pairs with similar genetic effects. Finally, we consider the two-locus models typically used in human genetics, the 50 models from [20], and some models with epistasis. We show that these models only represent a small fraction of all possible two-locus models.

## Results and Discussion

### Shapes of two-locus models

A two-locus disease model on two biallelic loci is specified by the genotype values of the 9 two-locus genotypes. We consider two loci with genotypes *aa, Aa*, and *AA*, and *bb, Bb*, and *BB*, respectively, where *A *and *B *are the susceptibility alleles. The genotype values, *f*_*ij*_, *i*, *j *= 0, 1, 2, are represented by a 3 × 3 table, where *i *and *j *refer to the number of disease alleles at loci *A *and *B*, respectively:

$$\begin{array}{cccc} & bb & Bb & BB\\ aa & f_{00} & f_{01} & f_{02}\\ Aa & f_{10} & f_{11} & f_{12}\\ AA & f_{20} & f_{21} & f_{22} \end{array}$$

In the case of a dichotomous trait, *f*_*ij *_can e.g. be a penetrance, the probability that an individual with genotype *ij *will get the disease. For a quantitative trait, *f*_*ij *_can e.g. be the expected phenotypic value for an individual with genotype *ij*.

In an additive model, the genotype values can be written as a sum of the effect at each locus, *f*_*ij *_= *α*_*i *_+ *β*_*j*_, where *α*_*i *_is the effect associated with having *i *disease alleles at the first locus, and *β*_*j *_is the effect associated with having *j *disease alleles at the second locus. An epistatic model is any non-additive two-locus model. To study epistasis we consider the interaction space, which is the space of all two-locus models modulo the space spanned by all additive two-locus models. The interaction space is spanned by a set of linear forms in the {*f*_*ij*_} called *circuits*. There is a circuit for each set of 3 collinear points, and for each set of four points in the plane such that no three of the points are collinear, resulting in a total of 86 circuits. The coefficients in the linear form are such that the sum of the points in the circuit, when scaled by these coefficients, is zero. For example, the circuit arising from the points *f*_00_, *f*_01_, *f*_20_, and *f*_12 _is

-3*f*_00 _+ 4*f*_01 _+ *f*_20 _- 2*f*_12_,

since

-3·(0, 0) + 4·(0, 1) + (2, 0) - 2·(1, 2) = 0.

Every circuit with four points can be seen as a contrast between two pairs of genotype values and measures a specific deviation from additivity. For example, the above circuit is positive if 4*f*_01 _+ *f*_20 _≥ 3*f*_00 _+ 2*f*_12 _and negative otherwise. For some circuits this contrast has a simple interpretation, e.g. the circuit arising from *f*_00_, *f*_01 _and *f*_02 _is *f*_00 _- 2*f*_01 _+ *f*_02_. It compares the genotype value *f*_01 _(for genotype *aa/Bb*) to the average of the genotypic values *f*_00 _and *f*_02 _(for genotypes *aa/bb *and *aa/BB*), i.e. it measures deviation from additivity at locus *B *in individuals with genotype *aa *at locus *A*.

To more easily interpret the meaning of the circuits we perform a change of coordinates. In quantitative trait genetics the phenotypic value is often decomposed into additive (*f*_*a *_and *f*_*b*_) and dominance (*δ*_*a *_and *δ*_*b*_) main effects at loci *A *and *B *respectively, and four epistatic effects, *additive × additive *(*I*_*AA*_), *additive × dominance *(*I*_*AD*_), *dominance × additive *(*I*_*DA*_), and *dominance × dominance *(*I*_*DD*_). We will use the same notation here to decompose the genotype values into main and epistatic effects. We write the two-locus model as

$$\begin{array}{cccc} & aa & Aa & AA\\ bb & \tilde{f}-f_{a}-f_{b}+I_{AA} & \tilde{f}+\delta_{a}-f_{b}-I_{AD} & \tilde{f}+f_{a}-f_{b}-I_{AA}\\ Bb & \tilde{f}-f_{a}+\delta_{b}-I_{DA} & \tilde{f}+\delta_{a}+\delta_{b}+I_{DD} & \tilde{f}+f_{a}+\delta_{b}+I_{DA}\\ BB & \tilde{f}-f_{a}+f_{b}-I_{AA} & \tilde{f}+\delta_{a}+f_{b}+I_{AD} & \tilde{f}+f_{a}+f_{b}+I_{AA} \end{array}$$

where

$$4\cdot \tilde{f}=f_{00}+f_{02}+f_{20}+f_{22}$$

4·*f*_*a *_= -*f*_00 _+ *f*_02 _- *f*_20 _+ *f*_22_

4·*f*_*b *_= -*f*_00 _- *f*_02 _+ *f*_20 _+ *f*_22_

4·*δ*_*a *_= -*f*_00 _+ 2*f*_01 _- *f*_02 _- *f*_20 _+ 2*f*_21 _- *f*_22_

4·*δ*_*b *_= -*f*_00 _+ 2*f*_10 _- *f*_20 _- *f*_02 _+ 2*f*_12 _- *f*_22_

4·*I*_*AA *_= *f*_00 _- *f*_02 _- *f*_20 _+ *f*_22_

4·*I*_*AD *_= *f*_00 _- 2*f*_01 _+ *f*_02 _- *f*_20 _+ 2*f*_21 _- *f*_22_

4·*I*_*DA *_= *f*_00 _- 2*f*_10 _+ *f*_20 _- *f*_02 _+ 2*f*_12 _- *f*_22_

4·*I*_*DD *_= *f*_00 _- 2*f*_01 _+ *f*_02 _- 2*f*_10 _+ 4*f*_11 _- 2*f*_12 _+ *f*_20 _- 2*f*_21 _+ *f*_22_

Note that with this choice the additive effect is scaled so that the contribution is -*f*_*a*_, 0, and *f*_*a *_for genotypes *aa*, *Aa*, and *AA *respectively, and similarly for the second locus. This is a simple linear transformation of the genotype values which can be used both for penetrances and phenotypic means. The space of all two-locus models has dimension 9 and the interaction space has dimension 6. A natural choice of a basis for the interaction space is given by the *interaction coordinates *(*δ*_*a*_, *δ*_*b*_, *I*_*AA*_, *I*_*AD*_, *I*_*DA*_, *I*_*DD*_) where *δ*_*a *_and *δ*_*b *_measure within-locus interaction and *I*_*AA*_, *I*_*AD*_, *I*_*DA*_, and *I*_*DD *_measure between-loci interaction.

A full list of the 86 circuits in the new coordinates is given in Appendix B. Although the circuits are fully specified by the six interaction coordinates they do contain important information on the type of interaction present. The circuits measure interesting contrasts and the new parameterization allows us to interpret them. For example, circuit *c*_30 _= 2*δ*_*a *_- 2*δ*_*b *_measures the difference between the dominance effects, circuit *c*_1 _= -2*δ*_*a *_+ 2*I*_*AD *_measures the difference between the dominance effect at the first locus and the *additive × dominance *interaction, etc. The sign of a circuit specifies whether the type of epistasis measured by the circuit is positive or negative, and its magnitude measures the degree of interaction. The circuits contain detailed information on the interaction in a model and to fully describe the pattern of interaction we can consider the sign pattern of all 86 circuits, however, this leads to a very large number of categories. For a more useful classification of all two-locus models according to the type of interaction present we consider the *triangulation *induced by the penetrances. The connection between a triangulation and the circuits will be discussed further below.

The mathematical definition of a triangulation is given in Appendix A but an informal description is provided here. We represent the 9 genotypes by 9 points in the plane on a 3 × 3 grid and the genotypic values by heights above these points. If the values come from an additive model it is possible to fit a plane through the height points. For any non-additive model we consider the surface given by the upper faces of the convex hull of the heights. Intuitively this is the surface formed if we were to drape a piece of stiff cloth on top of the heights and consider its shape. Any departure from additivity in the model becomes apparent in this surface. The triangulation, or *shape*, of a model is obtained by projecting these upper faces (the "creases" in the surface) onto the *xy*-plane.

**Figure 1:**
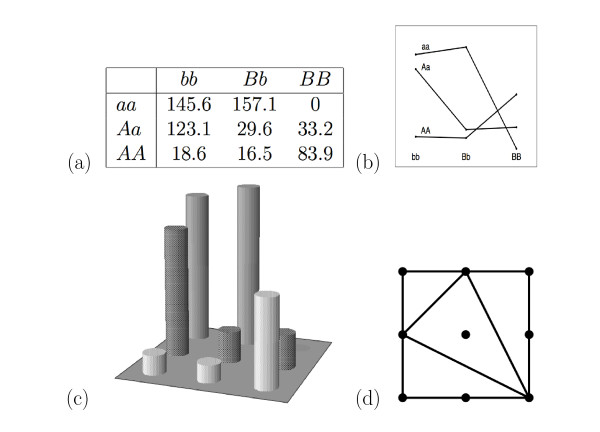
**Example of epistasis**. Example of epistasis in QTL data. The data is on chicken growth [24]. (a) The phenotypic means of the two-locus genotypes, (b) a wiggle plot of the data, where each line corresponds to a row in the table, (c) bar plot of the data, (d) the two-locus shape.

A visual representation of a two-locus model is given in Figure 1. The data comes from an example that will be discussed further later. The genotype values, relative to the value of *aa/BB*, are listed in Panel (a). Panel (b) shows the classical visualization of this table, where each line corresponds to one row in the table. In Panel (c) there is a bar-chart of the data, and the corresponding shape is shown in Panel (d). There is clearly epistatic interaction in the model in Figure 1, as the genotypes *aa/bb*, *aa/Bb*, *Aa/bb*, and *AA/BB *have much higher means than the remaining 5 genotypes. The shape shows the four planes of the upper convex hull of the heights. It includes a plane through the genotypes *Aa/bb*, *aa/Bb*, and *AA/BB*, which is given by the middle triangle in the picture, and three planes corresponding to the outer three triangles. Although the classical visualization in Panel (b) of Figure 1 contains complete information on the relative genotype values it is hard to grasp what types of interactions occur just by glancing at the figure. The bar-chart is a very good visual representation of the 9 values, however, any comparison between two different datasets based on bar-charts would be not only tedious, but hard to define. Some information is lost by considering only the shape of the model, but since it summarizes the epistasis that is present, the shape enables us to easily compare and classify different models.

**Figure 2:**
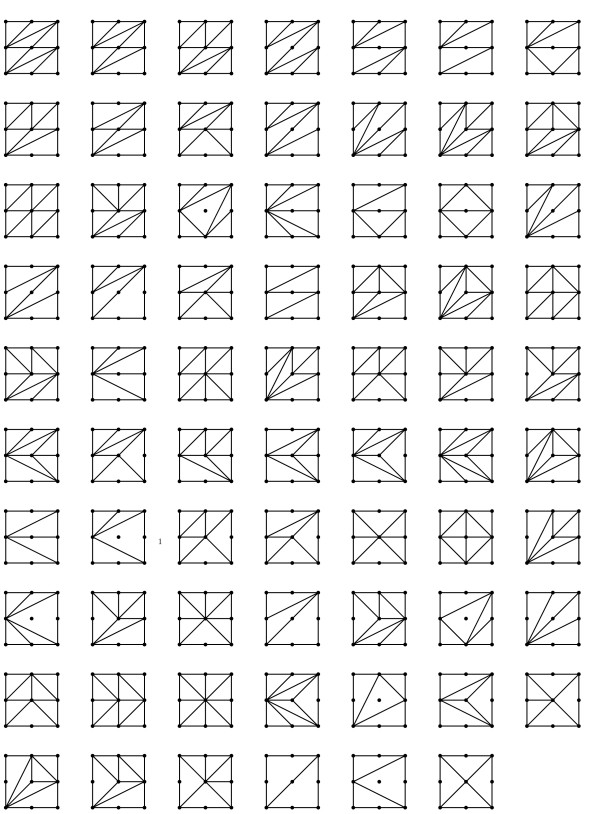
**Symmetry classes**. Shapes. The 69 symmetry classes of the shapes of two-locus models.

We used TOPCOM [21] to compute all possible triangulations, or shapes, and found that there are 387, however, many are equivalent when we account for symmetry. By symmetry we mean i) the interchange of locus 1 and locus 2, or ii) the interchange of two alleles at one or both loci. These same symmetry conditions were used in [20]. After accounting for symmetry, there are 69 shapes (see Figure 2). We classify all two-locus models according to which of the 387 (or 69) triangulations they belong to.

A sign pattern for the circuits specifies a model shape, but the converse is not true. Thus considering the shape of a model, rather than the sign pattern of the 86 circuits, gives a coarser model classification, but it provides a very useful description of the type of epistasis in the model. A shape contains information about the signs of some of the 86 circuits. Every group of points in a circuit can be triangulated in exactly two ways [22] corresponding to the type of epistasis. If a model shape has a line connecting the points (*i*_1_, *j*_1_) and (*i*_2_, *j*_2_) then for some circuit, $c=(a_{1}f_{i_{1}j_{1}}+a_{2}f_{i_{2}j_{2}})-(b_{1}f_{k_{1}l_{1}}+b_{2}f_{k_{2}l_{2}})$, the pair $f_{i_{1}j_{1}}$ and $f_{i_{2}j_{2}}$ are the "winners", i.e. $a_{1}f_{i_{1}j_{1}}+a_{2}f_{i_{2}j_{2}}\geq b_{1}f_{k_{1}l_{1}}+b_{2}f_{k_{2}l_{2}}$. Similarly, if there is no line connecting the points (*i*_1_, *j*_1_) and (*i*_2_, *j*_2_), and it is not possible to add one without crossing an existing line segment, then there is some circuit such that $f_{i_{1}j_{1}}$ and $f_{i_{2}j_{2}}$ are the "losers". For example, in Figure 1, there is a line between (1, 0) and (0, 1) and *f*_01 _+ *f*_10 _≥ *f*_00 _+ *f*_11_, and also 2*f*_01 _+ 2*f*_10 _≥ 3*f*_00 _+ *f*_22_.

Note that the model shape gives information about the types of interaction present in the model, but does not reveal the magnitude of the interaction (for that we need the actual value of the circuits). For generic models we always get a triangulation of the 3 × 3 grid, but for some models the resulting shape is not a triangulation but a subdivision, where not all cells in the shape are 3-sided (this happens e.g. when many of the genotype values are identical). These coarse subdivisions are not counted in our 387 models, however each coarse subdivision is refined by two or more of our models. The model shape provides information that is complementary to that given by the values of the interaction coordinates. Looking at a specific triangulation or subdivision tells us which way some (but not all) of the circuits are triangulated, thus giving information about interaction for that particular model, in particular the triangulation allows us to identify the dominating interactions. Consider e.g. the example in Figure 1. Although there is *additive × additive *interaction present (*I*_*AA *_= 210.9), and the circuit *c*_17 _= 4*I*_*AA *_is clearly positive, the corresponding line between (0, 0) and (2, 2) is not included. This is because this interaction is dominated by other types of interaction. The two circuits with the largest values are *c*_71 _and *c*_72_. The first of these contrasts *f*_01 _and *f*_22 _with *f*_02 _and *f*_10 _and thus the line between (0, 1) and (2, 2) is included in the triangulation, the second contrasts *f*_10 _and *f*_22 _with *f*_02 _and *f*_21 _and thus the line between (1, 0) and (2, 2) is included.

It is useful to have a notion of when two shapes are "close" or "similar". We say that two shapes are adjacent if one can move from one to the other by changing the sign of one of the 86 circuits (note that most sign changes do not result in a move between shapes). Out of 387 shapes, 350 are adjacent to 6 other shapes, 16 are adjacent to 7 other shapes, and 21 are adjacent to 8 other shapes. We define the distance between two shapes as the minimum number of circuit changes that are necessary to get from one to the other. In the set of 387 shapes the maximum distance between two shapes is 9, and around 70% of all pairs of shapes are distance 4 to 6 apart. Two-locus models which fall into adjacent model shapes share many of the same two-locus interactions, and in general the shorter the distance between two shapes, the more similar the genetic effects. For a further discussion on the shapes of genetic models see [23].

Each shape divides the 3 × 3 grid into 2 to 8 triangles (the numbers in each category are 2, 11, 38, 68, 96, 108 and 64 out of 387). Each shape corresponds to a subspace of 9-dimensional space and the volume of this subspace measures how much of the parameter space the shape inhabits. We obtained an estimate of this by generating 1,000,000 random vectors of length 9 and calculating the shape that each of them falls into. The model shape for a 9-vector is conserved under shifting and scaling so it suffices to consider vectors in [0, 1]^9 ^and each of the 9 numbers were drawn uniformly at random from the interval [0, 1]. The fraction of observations that fell into shapes which divide the grid into 2, 3, 4, triangles, etc., was 6.4%, 17.2%, 28.3%, 24.9%, 15.0%, 6.1% and 2.0%, very different from the fraction of shapes in each category, which is 0.5%, 2.8%, 9.8%, 17.6%, 24.9%, 29.9% and 16.5%. Two-locus models where one, or a few, genotype values are larger than the remaining values induce shapes which contain fewer triangles. However, if the genotype values show only slight deviations from falling on a plane (i.e. *δ*_*a*_, *δ*_*b*_, *I*_*AA*_, *I*_*AD*_, *I*_*DA*_, and *I*_*DD *_are small), the surface is not dominated by a few genotypes and the resulting shape will be more subdivided.

We will further discuss how the shapes can be used to characterize the type of interaction in a dataset using an example on QTL mapping in chicken.

### Two-locus models

In this section we study a number of model classes that are often used in genetic analysis, and the shapes that they induce. We show that each of the model classes restricts the analysis to a small subset of all possible two-locus models. Furthermore, because these models are very specific, they limit the types of interaction that can be modeled and only represent a small fraction of the 69 shapes.

A two-locus penetrance model can be defined by specifying single locus penetrance factors, (*α*_0_, *α*_1_, *α*_2_) and (*β*_0_, *β*_1_, *β*_2_), and combining them in one of three ways,

$$\begin{array}{c} multiplicative:f_{ij}=\alpha_{i}\cdot \beta_{j},\\ additive:f_{ij}=\min(\alpha_{i}+\beta_{j},1),\\ heterogeneous:f_{ij}=\alpha_{i}+\beta_{j}-\alpha_{i}\cdot \beta_{j}. \end{array}$$

**Figure 3:**
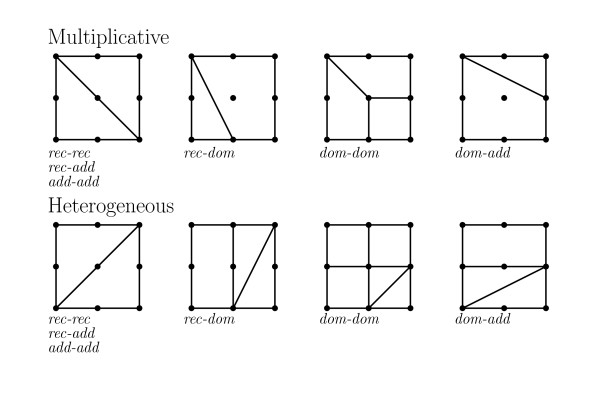
**Shapes of two-locus models**. Two-locus models. The model shapes for multiplicative and heterogeneous two-locus models.

The penetrance factors are typically chosen from a recessive (0, 0, *α*), dominant (0, *α*, *α*) or additive (0, *α*/2, *α*) model. For an additive two-locus model with additive penetrance factors, the interaction coordinates *δ*_*a*_, *δ*_*b*_, *I*_*AA*_, *I*_*AD*_, *I*_*DA*_, *I*_*DD *_are all zero and the circuits all vanish. For all additive two-locus models *I*_*AA *_= *I*_*AD *_= *I*_*DA *_= *I*_*DD *_= 0, but *δ*_*a *_and *δ*_*b *_depend on the penetrance factors. The heterogeneous model is often viewed as an approximation to the additive model because if the same penetrance factors are used, the models give very similar penetrances. However, in terms of the type of interaction that can be modeled, the multiplicative and heterogeneous models are very similar. In Table 1, we list the values of the interaction coordinates for some common multiplicative models. If we consider the corresponding heterogeneous models, the single locus dominance terms, *δ*_*a *_and *δ*_*b*_, have the same value, as listed in the table, and the interaction terms, *I*_*AA*_, *I*_*AD*_, *I*_*DA*_, and *I*_*DD*_, all have the same absolute value but opposite signs. The shapes induced by these models are shown in Figure 3. Note that only 8 shapes can be induced and 6 of the 8 shapes are not generic models (they are subdivisions rather than triangulations of the 3 × 3 grid).

**Table 1:** Classical two-locus models. The table lists the values of the interaction coordinates for multiplicative two-locus models. The parameters are *γ *= (*α*_2 _- *α*_0_)(*β*_2 _- *β*_0_), *η*_1 _= (*α*_0 _+ *α*_2_)(*β*_2 _- *β*_0_), and *η*_2 _= (*α*_2 _- *α*_0_)(*β*_0 _+ *β*_2_).

One-loc	*δ*_*a*_	*δ*_*b*_	*I*_*AA*_	*I*_*AD*_	*I*_*DA*_	*I*_*DD*_
*rec-rec*	-*η*_1_/4	-*η*_2_/4	*γ*	-*γ*	-*γ*	*γ*
*rec-add*	0	-*η*_2_	*γ*	0	0	-*γ*
*rec-dom*	-*η*_1_/4	-*η*_2_/4	*γ*	*γ*	-*γ*	-*γ*
*dom-dom*	-*η*_1_/4	-*η*_2_/4	*γ*	*γ*	*γ*	*γ*
*dom-add*	0	*η*_2_	*γ*	*γ*	0	0
*add-add*	0	0	*γ*	0	0	0

**Figure 4:**
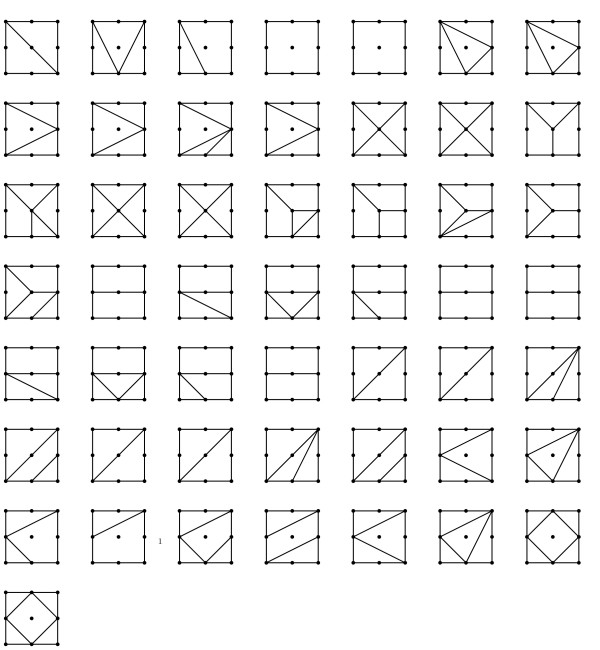
**Subdivisions**. 0/1 models. The subdivisions for the 50 Li and Reich 0/1 penetrance models.

In [20] a classification of all two-locus disease models with 0/1 penetrance values is given. Although this classification is useful to generate data under various scenarios and to study general properties of two-locus models, it cannot be used to classify observed data. This class of models is much larger than the class of disease models discussed above, yet they only cover a small part of all two-locus models. The 50 models represent only 29 unique subdivisions, and only 10 out of those 29 are among the 69 model shapes, see Figure 4.

**Figure 5:**
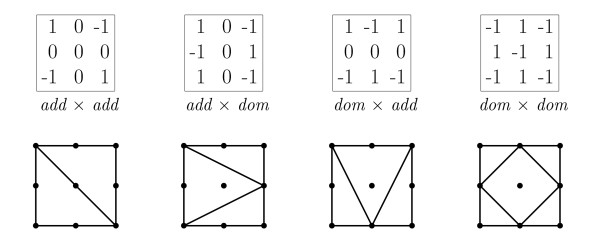
**Shapes of two-locus models**. Epistastic models. The tables list the genotype values associated with four epistatic models and below each table is the shape induced by a model with purely *add *× *add*, *add *× *dom*, *dom *× *add *or *dom *× *dom *interaction.

In population genetics and in the study of quantitative traits, two-locus models are classified according to the type of epistatic effects. Four commonly studied patterns of epistasis are *additive × additive*, *additive × dominance*, *dominance × additive *and *dominance × dominance*. In an *additive × additive *model two double homozygotes, *aa/bb *and *AA/BB*, have higher phenotypic mean (or fitness) than expected, but the other two, *aa/BB *and *AA/bb*, have lower phenotypic mean than expected. A numeric representation of the four types is given in Figure 5 and the corresponding shapes are also shown. If these epistatic effects are added to a fully additive two-locus model, the resulting shape will be the one shown in Figure 5. However, the epistasis observed in real data is seldom purely of one type and although e.g. *dominance × dominance *epistasis is present in the data, the resulting shape can be different. If the dominance terms, *δ*_*a *_and *δ*_*b*_, are non-zero, the resulting shape will be the *dominance × dominance *shape, with the possible addition of one or both of the horizontal and vertical lines through the middle of the shape (depending on the magnitude of the dominance terms). A model with both *additive × dominance *and *dominance × additive *interaction can fall into one of three shapes. If either the *additive × dominance *or the *dominance × additive *interaction is much stronger than the other, the corresponding shape will dominate. If the magnitude of both types of interaction is similar, the resulting shape will be the shape shown in Figure 1 or any rotation thereof. Thus from the shape we can often infer what type of interaction is the strongest in the data.

### Classification of epistatic effects

Model organisms such as yeast, mouse or chicken are frequently used in genetic analysis, and several recent studies have shown that epistatic effects contribute greatly to observed genetic variability. When pairs of interacting loci have been found, using either QTL mapping, linkage analysis, or association analysis, it is of interest to describe the epistasis in the data. If many pairs of interacting loci have been found, it is of interest to identify pairs with similar genetic effects. This classification can be based on finding, for each pair, the model which best fits the data, out of the classical two-locus models. However, many datasets do not fall into any one of these classes (e.g. more than one type of epistasis can be present in the data). Another option is to base the classification on visual inspection, but that can be inaccurate and very time consuming, especially since in most applications the two alleles at a locus are interchangeable, so one would have to consider many rotations of the 3 × 3 data matrix.

We propose classifying observations according to the shape that they induce, and measuring the similarity of the genetic effects observed in two different datasets by the minimum distance between their induced shapes. This allows us to quickly and automatically identify observations with similar genetic effects. Here we only consider the shape of a model for classification but a more robust classification, outside the scope of this paper, could be obtained by testing which circuits are non-zero and considering the shape induced after circuits which are not significant have been set to zero. This would help in reducing mis-classification due to measurement error in the data and in particular this would reveal whether the data comes from an additive (or near additive) model.

In a study of growth traits in chickens, [24] measured various growth and body weight variables on 546 chickens from an *F*_2 _cross between two lines, a commercial broiler sire line and a White Leghorn line. The alleles at each locus are labeled with *B *and *L*, according to which line they came from. A method for simultaneous mapping of interacting QTLs was used to do a genome-wide analysis of five growth traits which identified 21 QTL pairs with a significant genetic effect. Some of the 21 QTL pairs were associated with more than one growth trait, resulting in 30 combinations of traits and QTL pairs. For each trait and QTL pair the phenotypic means of each of the nine two-locus genotypes were estimated using linear regression (see Table 2 in [24]). They noted that the standard models for epistasis do not adequately describe the types of interaction present in their data, and classified the QTL pairs into groups with similar genetic effect by visual inspection. They identified 4 general classes of models in this dataset, and classified 16 out of the 21 QTL pairs into one of these classes (when a QTL pair was associated with more than one trait the observations from both traits were considered to be in the same class). The classes are H) some of the homozygote/heterozygote combinations are lower than expected, B) the phenotype value associated with the genotype BB/BB is lower than expected, A) the data fits an additive model, by visual inspection, L) there is a set of genotypes with a high value, a set with a low value associated with it, and the value associated with the genotype *LL/LL *is between the two, and U) the 5 QTL pairs which did not fit into any of the four classes were left unclassified.

**Figure 6:**
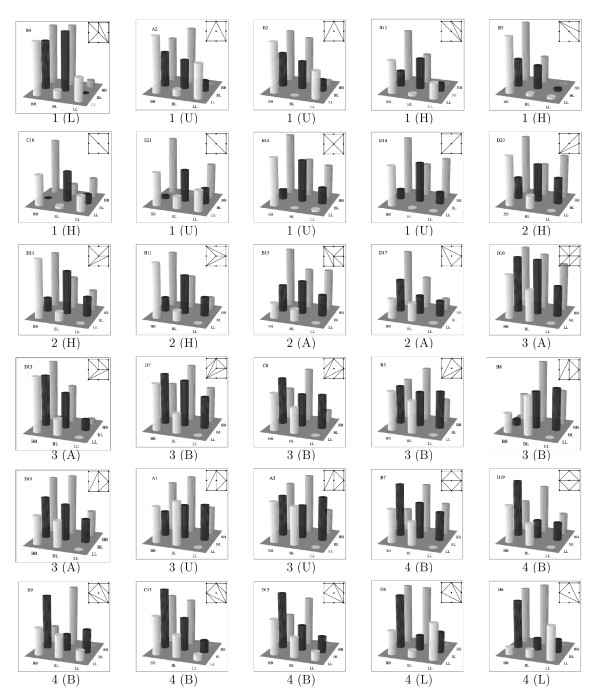
**Example**. Example of observed epistasis. A visual representation of the 30 trait/QTL pairs. The phenotype average for each genotype is given by the heights of the bars, the corresponding shape is also given, and the trait (A-E) and QTL pair (1–21) listed. Under each panel we list the cluster it falls into (1–4) and the group given by [24] (A, B, H, L, U).

We computed the shapes of the 30 observations and found that 23 of the 387 shapes occurred, or 16 out of 69 up to symmetry. The data are shown in Figure 6. For each observation we show a bar-chart of the phenotype means and the corresponding shape. The point in the upper left corner of the shape corresponds to the genotype *BB/BB*, and the point in the lower right corner corresponds to *LL/LL*. Although in most applications one would consider the two alleles at a locus to be interchangeable we do not here, since they come from different chicken lines. To group together observations with similar genetic effects we clustered the shapes based on the pair-wise distances between them, using complete linkage hierarchical clustering. There are four main clusters in the resulting dendrogram (not shown). Under each panel in Figure 6 we list which cluster it falls into, and in parentheses we list which group it belongs to according to [24]. The observations are ordered based on the hierarchical clustering with observations in the same cluster listed together and observations within each cluster listed according to the distance between them, as far as possible. For four observations we switched the order of the first and second locus, compared to the order in [24], in order to minimize the distance to the closest observation. Within a cluster, the distance between the shapes in side-by-side panels is typically one but occasionally two. Many of the observed shapes are adjacent to more than one other shape, so two shapes that are not adjacent in Figure 6 may still be close. Consider the last row in Figure 6. In all five panels the values of the genotypes *BB/BL*, *BL/BB *and *LL/LL *dominate the shape, resulting in a central triangular plane. The value at *BB/BB *varies considerably but does not affect the shape. The shape that each of the observations fall into is, however, affected by the values of *BL/LL *and *LL/BL*. When they are relatively high an additional partition is added in the shape. Recall from the previous section that this shape is observed when there is both *additive × dominance *and *dominance × additive *interaction in the data. The shapes in the second-to-last row indicate strong *dominance × dominance *interaction (compare to the shape given in Figure 5). In the last two observations in the row, *dom × dom *is the strongest interaction, whereas the first three also show strong *add × dom *interaction.

The visual classification corresponds very well to the classification based on shapes. All observations labeled H fall into clusters 1 and 2 (which are close to each other in the dendrogram) and all observations labeled B fall into clusters 3 and 4. The observations in group A (additive model) fall into two different clusters. An additive model has no shape (one can fit a plane through the points) but due to measurement error in real data this will not be the case. Note that 3 of the 5 observations in group A induce shapes which are very subdivided, as can be expected when there are no genotypes with very high values which dominate the shape. The observations in group U, which were previously unclassified, have now been grouped with the observations they are closest to. Two QTL pairs (4 and 6) were grouped together in category L. The two observations on QTL pair 6 are in cluster 4 and the observation on QTL pair 4 in cluster 1.

### The power to detect epistasis depends on the model shape

In the previous sections we have discussed how to visualize and classify interaction, however, the first step in a two-locus analysis is typically to identify pairs of loci with statistically significant interaction. We now ask whether (and how) the power to detect interaction depends on the true model shape. To fully answer that question it is necessary to perform a thorough simulation study which is outside the scope of this paper, but we have performed a preliminary analysis with the goal of comparing the relative power to detect interaction under different model shapes. We considered three different situations: QTL mapping, association analysis using logistic regression, and association analysis using an LD based measure for interaction. We consider the power to detect interacting loci as a function of only the true model shape although the power will also depend on the minor allele frequency at the two loci, the sample size in the study, the number of genotyped markers, and the prevalence of disease/phenotypic mean in the population. In two-locus QTL mapping, the phenotype is typically modeled as a function of the genotype using a linear model. If *y *is the phenotype, the model is

$$\begin{array}{lll} y & = & \tilde{f}+f_{a}\cdot x_{A}+f_{b}\cdot x_{B}+\delta_{a}\cdot x_{Aa}+\delta_{b}\cdot x_{Bb}\\ & & +I_{AA}\cdot x_{A}x_{B}+I_{AD}\cdot x_{Aa}x_{B}+I_{DA}\cdot x_{A}x_{Bb}+I_{DD}\cdot x_{Aa}x_{BB}+\varepsilon , \end{array}$$

where the coefficients of the model are the coordinates $\tilde{f}$, *f*_*a*_, *f*_*b*_, *δ*_*a*_, *δ*_*b*_, *I*_*AA*_, *I*_*AD*_, *I*_*DA *_and *I*_*DD*_, and *ε *is Gaussian. The x_* _are dummy variables; *x*_*A *_takes the values -1, 0, and 1 for individuals with genotypes *aa*, *Aa*, and *AA*, respectively, and *x*_*Aa *_takes the value 1 for individuals with genotype *A*_*a*_. The variables *x*_*B *_and *x*_*Bb *_are defined similarly. To test for epistasis, the fit of this model is compared to an additive model where *I*_*AA *_= *I*_*AD *_= *I*_*DA *_= *I*_*DD *_= 0. The test statistic for a likelihood ratio test is minus twice the difference between the log-likelihood of the additive and the full model. This is equivalent to testing if the circuits *c*_7 _= *c*_8 _= *c*_9 _= *c*_10 _= 0.

In case-control association studies the penetrances of the genotypes are not observed, only the counts of cases and controls that have each genotype. To study the shape of a two-locus disease model we can fit a full two-locus model using logistic regression and obtain the fitted log odds-ratio for each genotype, which can then be used to obtain an estimate of the penetrances. The model is:

$$\begin{array}{lll} \log\left(\frac{f_{ij}}{1-f_{ij}}\right) & = & \tilde{f}+f_{a}\cdot x_{A}+f_{b}\cdot x_{B}+\delta_{a}\cdot x_{Aa}+\delta_{b}\cdot x_{Bb}+I_{AA}\cdot x_{A}x_{B}\\ & & +I_{AD}\cdot x_{Aa}x_{B}+I_{DA}\cdot x_{A}x_{Bb}+I_{DD}\cdot x_{Aa}x_{BB}+\varepsilon , \end{array}$$

where the *f*_*ij *_are penetrances and the dummy variables x_* _are defined as above. By using logistic regression the log-odds scale is chosen as the scale of interest, and additivity on that scale corresponds to no interaction. A likelihood ratio test for epistasis compares the fit of the full model to an additive model where *I*_*AA *_= *I*_*AD *_= *I*_*DA *_= *I*_*DD *_= 0. This test is equivalent to testing *c*_7 _= *c*_8 _= *c*_9 _= *c*_10 _= 0 where the circuits are obtained by replacing *f*_*ij *_with log(*f*_*ij*_/(1 - *f*_*ij*_)).

Recently [10] proposed a new test to detect unlinked interacting disease loci. They use an LD based interaction measure, *I *= *h*_00_*h*_11 _- *h*_01_*h*_10_, where *h*_*ij *_is defined as the penetrance of a haplotype *h*_*ij *_(*h*_00 _is the haplotype *ab*, *h*_01 _is *aB*, etc.). The haplotype penetrance depends on the two locus penetrances as well as the allele frequencies. It is easy to show that the interaction measure, *I*, vanishes if *c*_7 _= *c*_8 _= *c*_9 _= *c*_10 _= 0 when the circuits are calculated using the log penetrance values. In other words, this interaction measure tests for multiplicative penetrances.

**Figure 7:**
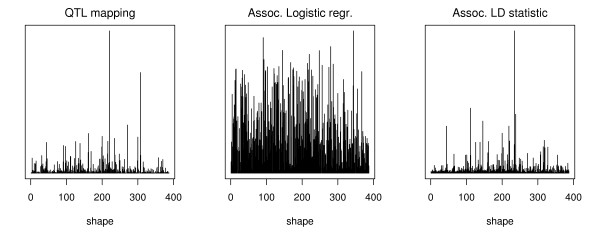
**Power to detect association**. Power to detect association. The plots show the maximum value of the likelihood ratio test statistic observed for randomly generated data from each of the 387 shapes.

We generated 50, 000 random vectors of length 9. For the QTL analysis we fixed the population mean of the phenotype, fixed the allele frequencies of *A *and *B*, and then normalized each random vector to give the desired population mean. For each vector we generated 10 datasets, each with sample size 300, and fit both the full model and an additive model (note that for all of the models the 6 interaction coordinates are non-zero so the tests all have the same degrees of freedom). We used the average likelihood ratio statistic as an indicator of the power to detect interaction for that particular model. For each random model we then recorded which of the 387 model shapes it fell into and for each shape looked at maximum of the likelihood ratio statistic. In the first panel of Figure 7 we show the maximum for each shape. These maxima are highly variable between shapes, indicating that some types of interactions are easier to detect than others. We also observed that there is a strong association between large values of the likelihood ratio test statistic and the number of polygons a shape divides the square into.

We also generated case-control data from an association study. The random vectors were normalized so that they all give the same population prevalence of disease. In the middle panel of Figure 7 we have plotted the maximum of the likelihood ratio test statistic as a function of the shape induced by the penetrances. The test measures deviation from additivity on a log-odds scale, so the difference between the different shapes is relatively small. When the shape is calculated based on the log-odds the results are the same as before. Finally, in Panel 3 of Figure 7 we plot the maximum absolute value of the interaction measure *I*. This test measures deviation from additivity on a log scale, yet the results seem to be more similar to the QTL mapping case.

## Conclusion

The multitude of terms used to describe gene interactions are a testament not only to the importance of describing and classifying gene interaction, but also to the fact that even in a two-locus model the types of interactions that can and do occur are diverse and difficult to classify. Most examples of gene interactions that are observed in real data do not fall into any one of the categories typically used to describe interactions. Our approach overcomes this limitation and provides a complete classification of all two-locus models with continuous genotypic values into 69 (or 387) classes. The shape of a two-locus model reveals information about the types of gene interaction present and provides a visual representation of epistasis. By comparing an observed shape to the shapes of standard epistatic models we see which type of interaction is strongest in the data. Moreover, the values of the individual circuits listed in Appendix B provide a complete description of the epistasis in a two-locus system. The observed shape can differ from the true underlying model shape due to noise in the data. Rather than assign an observation to a shape based on the observed genotype values, one could test which circuits are significantly different from zero and use only those circuits to obtain the shape.

Two-locus models are frequently used to generate simulated datasets that form the basis for studies of the power of single-locus and two-locus methods. These can then be used e.g. to choose between exhaustive two-locus searches or two-stage two-locus analyses. There are many examples, both for linkage analysis and association analysis, where the results and ensuing recommendations depend on the models, and types of gene interactions, that are considered [8,9]. With our complete classification it is possible to generate data from each model class (while varying parameters such as population prevalence and allele frequencies) and subsequently a more thorough analysis than previously possible can be performed.

As observed in [24] "there are no striking similarities with a Mendelian pattern of digenic epistasis" in the QTL example and we found many types of nontrivial interaction, including models which cannot easily be described using existing models. The fact that our classification is purely mathematical lends it strength, since we can describe all possible models and categorize them according to the relative genotypic values. It can easily be extended to three or more loci. It remains to be seen whether all of the 69 types occur in nature. Our results also provide a formalism for identifying types of epistasis that may play a role in determining genetic variability in populations [25], but we do not address these implications in this paper.

## Authors' contributions

IBH and DSY both analysed the data, wrote the software and prepared the manuscript. DSY studied the triangulations of the two-locus model and IBH worked on the connections to biology. IBH also guided the project. Both authors read and approved the manuscript.

## Appendix A: Polyhedral subdivisions

Our classification is based on the theory of *regular polyhedral subdivisions*.

**Definition 1 ***A polyhedral subdivision of a point set A is a decomposition of conv*(*A*), *the convex hull of A, into a finite number of bounded polyhedra, such that the union of these polyhedra is conv*(*A*), *and the intersection of any two polyhedra is a common face of each (possibly the empty face)*.

A polyhedral subdivision where all the polyhedra are simplices is called a *triangulation*. We can construct a *regular *polyhedral subdivision of a point set *A *using the following construction: Assign to every point *a*_*i *_in *A *a 'height', *h*_*i*_. Then lift each point in *A *to its specified height by forming the new point set

$$\tilde{A}={\left\{(a_{i},h_{i})\right\}}_{a\in A}.$$

Take *conv*($\tilde{A}$), and consider its "upper faces", that is, the faces whose outward-pointing normal vector has its last coordinate positive. Project each upper face onto *conv*(*A*), by dropping the final coordinate of each point. In this manner, we obtain a polyhedral subdivision of *A*. Note that some points of *A *may not be used in this subdivision.

**Remark 2 ***In the construction of an induced subdivision there is some ambiguity as to the whether to project with the lower or upper faces of conv*($\tilde{A}$). Both conventions are commonplace. We chose to use the upper faces in order to stay consistent with literature on induced subdivisions and gene epistasis [19].

If the set of heights {*h*_*i*_} is sufficiently generic, then the subdivision induced by the heights will be a triangulation. We will only consider regular subdivisions and triangulations, thus we will use the term "subdivision" to mean "regular polyhedral subdivision", and "triangulation" to mean "regular triangulation". For more on polyhedral geometry see the book [26].

## Appendix B: Circuits

*c*_1 _= -2*δ*_*a *_+ 2*I*_*AD*_

*c*_2 _= -2*δ*_*a *_- 2*I*_*DD*_

*c*_3 _= -2*δ*_*a *_- 2*I*_*AD*_

*c*_4 _= -2*δ*_*b *_+ 2*I*_*DA*_

*c*_5 _= -2*δ*_*b *_- 2*I*_*DD*_

*c*_6 _= -2*δ*_*b *_- 2*I*_*DA*_

*c*_7 _= *I*_*AA *_+ *I*_*AD *_+ *I*_*DA *_+ *I*_*DD*_

*c*_8 _= *I*_*AA *_- *I*_*AD *_+ *I*_*DA *_- *I*_*DD*_

*c*_9 _= *I*_*AA *_- *I*_*AD *_- *I*_*DA *_+ *I*_*DD*_

*c*_10 _= *I*_*AA *_+ *I*_*AD *_- *I*_*DA *_- *I*_*DD*_

*c*_11 _= -2*δ*_*a *_- 2*δ*_*b *_+ 2*I*_*AA *_- 2*I*_*DD*_

*c*_12 _= -2*δ*_*a *_- 2*δ*_*b *_- 2*I*_*AA *_- 2*I*_*DD*_

*c*_13 _= 2*I*_*AA *_+ 2*I*_*DA*_

*c*_14 _= 2*I*_*AA *_- 2*I*_*DA*_

*c*_15 _= 2*I*_*AA *_+ 2*I*_*AD*_

*c*_16 _= 2*I*_*AA *_- 2*I*_*AD*_

*c*_17 _= 4*I*_*AA*_

*c*_18 _= -2*δ*_*a *_+ *I*_*AA *_+ *I*_*DA *_- *I*_*DD *_+ *I*_*AD*_

*c*_19 _= 2*δ*_*a *_+ *I*_*AA *_+ *I*_*DA *_+ *I*_*DD *_- *I*_*AD*_

*c*_20 _= -2*δ*_*a *_+ *I*_*AA *_- *I*_*DA *_- *I*_*DD *_- *I*_*AD*_

*c*_21 _= 2*δ*_*a *_+ *I*_*AA *_- *I*_*DA *_+ *I*_*DD *_+ *I*_*AD*_

*c*_22 _= -2*δ*_*b *_+ *I*_*AA *_+ *I*_*DA *_- *I*_*DD *_+ *I*_*AD*_

*c*_23 _= -2*δ*_*b *_- *I*_*AA *_+ *I*_*DA *_- *I*_*DD *_- *I*_*AD*_

*c*_24 _= -2*δ*_*b *_+ *I*_*AA *_- *I*_*DA *_- *I*_*DD *_- *I*_*AD*_

*c*_25 _= -2*δ*_*b *_- *I*_*AA *_- *I*_*DA *_- *I*_*DD *_+ *I*_*AD*_

*c*_26 _= -2*δ*_*a *_+ 2*I*_*AA*_

*c*_27 _= 2*δ*_*a *_+ 2*I*_*AA*_

*c*_28 _= -2*δ*_*b *_+ 2*I*_*AA*_

*c*_29 _= -2*δ*_*b *_- 2*I*_*AA*_

*c*_30 _= 2*δ*_*a *_- 2*δ*_*b*_

*c*_31 _= 2*δ*_*a *_+ 2*I*_*AA *_+ 2*I*_*DA *_+ 2*I*_*DD*_

*c*_32 _= -2*δ*_*a *_+ 2*I*_*AA *_+ 2*I*_*DA *_- 2*I*_*DD*_

*c*_33 _= 2*δ*_*a *_+ 2*I*_*AA *_- 2*I*_*DA *_+ 2*I*_*AD*_

*c*_34 _= -2*δ*_*a *_+ 2*I*_*AA *_- 2*I*_*DA *_- 2*I*_*AD*_

*c*_35 _= -2*δ*_*b *_- 2*I*_*AA *_- 2*I*_*DD *_+ 2*I*_*AD*_

*c*_36 _= 2*δ*_*b *_+ 2*I*_*AA *_+ 2*I*_*DD *_+ 2*I*_*AD*_

*c*_37 _= -2*δ*_*a *_+ 2*I*_*AA *_+ 2*I*_*DA *_+ 2*I*_*AD*_

*c*_38 _= 2*δ*_*a *_+ 2*I*_*AA *_+ 2*I*_*DA *_- 2*I*_*AD*_

*c*_39 _= -2*δ*_*b *_+ 2*I*_*AA *_- 2*I*_*DD *_+ 2*I*_*AD*_

*c*_40 _= 2*δ*_*b *_+ 2*I*_*AA *_+ 2*I*_*DA *_- 2*I*_*AD*_

*c*_41 _= -2*δ*_*b *_+ 2*I*_*AA *_- 2*I*_*DD *_- 2*I*_*AD*_

*c*_42 _= -2*δ*_*b *_- 2*I*_*AA *_+ 2*I*_*DA *_- 2*I*_*AD*_

*c*_43 _= 2*δ*_*a *_+ 2*I*_*AA *_- 2*I*_*DA *_+ 2*I*_*DD*_

*c*_44 _= -2*δ*_*b *_+ 2*I*_*AA *_+ 2*I*_*DA *_+ 2*I*_*AD*_

*c*_45 _= -2*δ*_*b *_+ 2*I*_*AA *_- 2*I*_*DA *_- 2*I*_*AD*_

*c*_46 _= -2*δ*_*a *_+ 2*I*_*AA *_- 2*I*_*DA *_- 2*I*_*DD*_

*c*_47 _= -2*δ*_*a *_+ 4*I*_*AA *_+ 2*I*_*AD*_

*c*_48 _= -2*δ*_*b *_+ 4*I*_*AA *_- 2*I*_*DA*_

*c*_49 _= -2*δ*_*a *_+ 4*I*_*AA *_- 2*I*_*AD*_

*c*_50 _= 2*δ*_*a *_+ 4*I*_*AA *_- 2*I*_*AD*_

*c*_51 _= -2*δ*_*b *_- 4*I*_*AA *_+ 2*I*_*DA*_

*c*_52 _= 2*δ*_*a *_+ 4*I*_*AA *_+ 2*I*_*AD*_

*c*_53 _= 2*δ*_*b *_+ 4*I*_*AA *_+ 2*I*_*DA*_

*c*_54 _= -2*δ*_*b *_+ 4*I*_*AA *_+ 2*I*_*DA*_

*c*_55 _= -2*δ*_*a *_+ 2*δ*_*b *_+ 2*I*_*DA *_+ 2*I*_*AD*_

*c*_56 _= 2*δ*_*a *_- 2*δ*_*b *_+ 2*I*_*DA *_+ 2*I*_*AD*_

*c*_57 _= 2*δ*_*a *_- 2*δ*_*b *_+ 2*I*_*DA *_- 2*I*_*AD*_

*c*_58 _= 2*δ*_*a *_- 2*δ*_*b *_- 2*I*_*DA *_+ 2*I*_*AD*_

*c*_59 _= 2*δ*_*a *_+ 2*δ*_*b *_+ 4*I*_*AA *_+ 2*I*_*DA *_- 2*I*_*AD*_

*c*_60 _= -2*δ*_*a *_- 2*δ*_*b *_+ 4*I*_*AA *_- 2*I*_*DA *_- 2*I*_*AD*_

*c*_61 _= -2*δ*_*a *_- 2*δ*_*b *_+ 4*I*_*AA *_+ 2*I*_*DA *_+ 2*I*_*AD*_

*c*_62 _= -2*δ*_*a *_- 2*δ*_*b *_- 4*I*_*AA *_+ 2*I*_*DA *_- 2*I*_*AD*_

*c*_63 _= 2*δ*_*a *_- 4*δ*_*b *_- 2*I*_*AA *_- 2*I*_*DA *_+ 2*I*_*AD*_

*c*_64 _= 4*δ*_*a *_- 2*δ*_*b *_+ 2*I*_*AA *_+ 2*I*_*DA *_- 2*I*_*AD*_

*c*_65 _= -4*δ*_*a *_+ 2*δ*_*b *_+ 2*I*_*AA *_+ 2*I*_*DA *_+ 2*I*_*AD*_

*c*_66 _= 2*δ*_*a *_- 4*δ*_*b *_- 2*I*_*AA *_+ 2*I*_*DA *_- 2*I*_*AD*_

*c*_67 _= 2*δ*_*a *_- 4*δ*_*b *_+ 2*I*_*AA *_- 2*I*_*DA *_- 2*I*_*AD*_

*c*_68 _= 4*δ*_*a *_- 2*δ*_*b *_+ 2*I*_*AA *_- 2*I*_*DA *_+ 2*I*_*AD*_

*c*_69 _= 2*δ*_*a *_- 4*δ*_*b *_+ 2*I*_*AA *_+ 2*I*_*DA *_+ 2*I*_*AD*_

*c*_70 _= 4*δ*_*a *_- 2*δ*_*b *_- 2*I*_*AA *_+ 2*I*_*DA *_+ 2*I*_*AD*_

*c*_71 _= 4*δ*_*a *_- 2*δ*_*b *_+ 4*I*_*AA *_+ 2*I*_*DA *_- 4*I*_*AD*_

*c*_72 _= 4*δ*_*a *_- 2*δ*_*b *_- 4*I*_*AA *_+ 2*I*_*DA *_+ 4*I*_*AD*_

*c*_73 _= 4*δ*_*a *_- 2*δ*_*b *_+ 4*I*_*AA *_- 2*I*_*DA *_+ 4*I*_*AD*_

*c*_74 _= 2*δ*_*a *_- 4*δ*_*b *_- 4*I*_*AA *_+ 4*I*_*DA *_- 2*I*_*AD*_

*c*_75 _= -2*δ*_*a *_+ 4*δ*_*b *_+ 4*I*_*AA *_+ 4*I*_*DA *_- 2*I*_*AD*_

*c*_76 _= -4*δ*_*a *_+ 2*δ*_*b *_+ 4*I*_*AA *_+ 2*I*_*DA *_+ 4*I*_*AD*_

*c*_77 _= 2*δ*_*a *_- 4*δ*_*b *_+ 4*I*_*AA *_+ 4*I*_*DA *_+ 2*I*_*AD*_

*c*_78 _= 2*δ*_*a *_- 4*δ*_*b *_+ 4*I*_*AA *_- 4*I*_*DA *_- 2*I*_*AD*_

*c*_79 _= -4*δ*_*a *_- 2*δ*_*b *_- 2*I*_*DA *_- 4*I*_*DD*_

*c*_80 _= -2*δ*_*a *_- 4*δ*_*b *_- 4*I*_*DD *_+ 2*I*_*AD*_

*c*_81 _= -4*δ*_*a *_- 2*δ*_*b *_+ 2*I*_*DA *_- 4*I*_*DD*_

*c*_82 _= -2*δ*_*a *_- 4*δ*_*b *_- 4*I*_*DD *_- 2*I*_*AD*_

*c*_83 _= -2*δ*_*a *_- 2*δ*_*b *_+ *I*_*AA *_+ *I*_*DA *_- 3*I*_*DD *_+ *I*_*AD*_

*c*_84 _= -2*δ*_*a *_- 2*δ*_*b *_- *I*_*AA *_+ *I*_*DA *_- 3*I*_*DD *_- *I*_*AD*_

*c*_85 _= -2*δ*_*a *_- 2*δ*_*b *_+ *I*_*AA *_- *I*_*DA *_- 3*I*_*DD *_- *I*_*AD*_

*c*_86 _= -2*δ*_*a *_- 2*δ*_*b *_- *I*_*AA *_- *I*_*DA *_- 3*I*_*DD *_+ *I*_*AD*_
